# Mosquito (Diptera: Culicidae) Diversity and Community Structure in Doi Inthanon National Park, Northern Thailand

**DOI:** 10.3390/insects13090814

**Published:** 2022-09-07

**Authors:** Wichai Srisuka, Chayanit Sulin, Wirat Sommitr, Rampa Rattanarithikul, Kittipat Aupalee, Atiporn Saeung, Ralph E. Harbach

**Affiliations:** 1 Entomology Section, Queen Sirikit Botanic Garden, P.O. Box 7, Chiang Mai 50180, Thailand; 2 Museum of World Insects and Natural Wonders, Chiang Mai 50200, Thailand; 3 Center of Insect Vector Study, Department of Parasitology, Faculty of Medicine, Chiang Mai University, Chiang Mai 50200, Thailand; 4 Scientific Associate, Natural History Museum, London SW7 5BD, UK

**Keywords:** distribution, ecology, mosquito fauna, mountain, vectors

## Abstract

**Simple Summary:**

Mosquitoes are small flies, some of which are known as principal vectors of the pathogens that cause dengue, malaria, filariasis, chikungunya, Japanese encephalitis, and Zika in many countries of the world. However, the majority of the species are important components of ecosystems. We evaluated the diversity and community structure of mosquitoes in Doi Inthanon National Park, named for the highest mountain in Thailand. The park mainly consists of natural forest, but it hosts a number of human activities. A total of 140 mosquito species were identified among 3,795 specimens. The most dominant genera were *Culex* (34.3%), *Aedes* (19.3%), *Anopheles* (13.6%), *Uranotaenia* (10.7%), and *Armigeres* (5.8%). The five most abundant species were *Ae.* *albopictus*, *Ae. vittatus*, *Cx. bitaeniorhynchus*, *Cx. mimulus*, and *Tripteroides aranoides*. Species richness was higher in the rainy season than in the cold and hot seasons. Natural phytotelm habitats for immature stages had a higher diversity than artificial container habitats. A greater number of species were found in forest areas than in agricultural areas and villages. Ground pools, stream pools, rock pools, bamboo stumps, bamboo internodes, and rice fields were the most preferred natural habitats. Ground pools, rock pools, and stream margins, which had a high diversity index, were the most important habitats for mosquitoes in the park.

**Abstract:**

Urbanization and human activities create new suitable aquatic habitats for the immature stages of mosquitoes in many countries. This also applies to Doi Inthanon National Park in northern Thailand, which is named for the highest mountain in the country. Despite its popularity, there is no information regarding mosquito diversity and community structure in the different ecosystems of the park. Monthly collections of immature stages from various habitats were conducted from August 2004 to December 2005 using dipping and sucking methods. The specimens collected from each habitat were reared to adults and identified based on their morphology. Diversity parameters and community structure were statistically analyzed. A total of 140 species (3795 specimens) belonging to 15 genera were identified. Among these, four genera (*Culex*, *Aedes*, *Anopheles*, and *Uranotaenia*) had high species richness, each represented by 48, 27, 19, and 15 species, respectively. *Aedes albopictus* was the most relatively abundant species, representing 6.7% of the total number of captured specimens, followed by *Tripteroides aranoides* (5.6%) and *Cx. mimulus* (5%). Species richness in natural habitats was significantly higher than in artificial containers. Species richness and abundance were highest in the rainy season. In comparison to agricultural areas and villages, mosquito diversity was found to be higher in forest areas. Ground pools, stream pools, rock pools, bamboo stumps, bamboo internode, and rice fields were the most preferred natural habitats. The results indicate that Doi Inthanon National Park has a high mosquito diversity. Each species exhibits differences in abundance and distribution in different habitats, which is useful information for planning conservation measures and vector control in the park.

## 1. Introduction

Mosquitoes are insects of major public health concern, mainly for the role of some species as vectors of pathogens of human and animal diseases, including the pathogens that cause dengue, chikungunya, Zika, Japanese encephalitis, West Nile fever, yellow fever, and malaria in humans. Approximately 60 pathogens can be transmitted to animals through mosquito bites [1,2,3]. Only female mosquitoes are of medical importance. Their bites can cause pain, itching, and red bumps, and scratching those bites can cause bacterial infections [4]. In 2020, there were 105,088 cases of the four main diseases in Thailand, i.e., dengue fever (90,915 cases), malaria (4473 cases), chikungunya fever (14,100 cases), and Zika fever (155 cases) [5]. However, not all mosquitoes are vectors (less than 5% of all mosquitoes in the world), and most of them are important components in aquatic and terrestrial ecosystems [4,6,7]. For example, nonbiting mosquitoes in the genus *Toxorhynchites* are beneficial because they can be used for biological control and to study virus propagation. The larval stages of *Toxorhynchites* are natural predators of other mosquito larvae (e.g., *Aedes aegypti*, *Ae*. *albopictus*, *Culex quinquefasciatus*, and *Cx*. *vishnui*) and aquatic organisms that live in the same phytotelm habitats [3,8]. In addition, *Ae*. *aegypti*, *Ae. communis*, and *Ae. canadensis* play a role as pollinators of *Platanthera obtusata* (an orchid) while feeding on the nectar of the flowers [9,10,11,12].

Mosquitoes are widely distributed in temperate, subtropical, and tropical regions of the world and well beyond the Arctic Circle. More than 3600 species in 41 genera (traditional classification; 113 genera are included with acceptance of the phylogenetic classification of the tribe Aedini) have been recognized worldwide [13]. In Thailand, the distribution of mosquitoes ranges from the summit of the highest mountain (Doi Inthanon) to the mangroves in lowland coastal areas. As a result of taxonomic studies conducted since Rattanarithikul et al. [14], who recognized 459 species in Thailand, the number of species known to occur in the country has increased to 466 [15,16,17,18,19,20,21]. Several species are considered to be of medical importance, such as *Aedes aegypti*, *Ae. albopictus*, *Anopheles aconitus*, *An. annularis*, *An. baimaii*, *An. barbirostris*, *An. culicifacies*, *An. dirus*, *An. maculatus*, *An. minimus*, *An. nivipes*, *An. pseudowillmori*, *An. sawadwongporni*, *An. stephensi*, *An. tessellatus*, *Culex quinquefasciatus*, *Cx. vishnui*, and *Mansonia uniformis* [3,4,22]. Their larval habitats are natural bodies of water (e.g., leaf axils, tree holes, rock holes, crab holes, bromeliads, pitcher plants, bamboo stumps, bamboo internodes, and rice fields) and artificial habitats (e.g., cans, cement tanks, plastic buckets, and tires) [1,23].

Mosquitoes are most diverse in tropical forest environments [13], where the warm moist climate is favorable for rapid development and adult survival, and the diversity of habitats fosters the evolution of many species [24]. Mosquitoes are very sensitive and quickly adapt to habitats and climate changes, and forest structure. These factors permit some species to develop in new habitats, such as artificial containers, or spread to areas that were previously unsuitable [4,25,26,27,28,29,30,31]. Several studies on the diversity, distribution, and ecology of mosquitoes in some countries have been published [7,31,32,33,34,35,36,37,38]. In Thailand, ecological studies have focused on only vector species, such as malaria vectors in some regions of the country [39,40,41,42,43,44,45,46,47,48].

Doi Inthanon National Park is well known as “the roof of Thailand”. The park is named for the highest mountain in the country (elevation 2565 m), and covers an area of 482.4 km^2^. It is a far eastern part of the Himalayan mountain range, with 2000 mm of annual precipitation and an average annual temperature of 20 °C (0–28 °C) [49]. The park has diverse microhabitats and unique climates depending on elevation, which ranges between 400 and 2,565 m above sea level. Most areas of higher elevation include several forest types, especially cloud forest, which has high moisture throughout the year. There are numerous plant species and the animal fauna includes 65 species of mammals and 370 bird species [49,50,51,52], and many groups of insects [53,54,55,56,57]. The park has numerous permanent and seasonally temporary streams and diverse other aquatic habitats, which serve as natural habitats for the immature stages of mosquitoes [4,58]. However, human activities, including urbanization, agriculture, and tourism, are the main cause of decreasing natural habitats, while new habitats are being created, some especially suitable for vector species [59,60,61,62,63,64,65].

Due to a lack of data on the diversity and community structure of mosquitoes in tropical forest ecosystems in Thailand, this study aimed to evaluate the diversity and community structure of mosquitoes across different ecosystems in Doi Inthanon National Park. 

## 2. Material and Methods

### 2.1. Study Area

**Figure 1:**
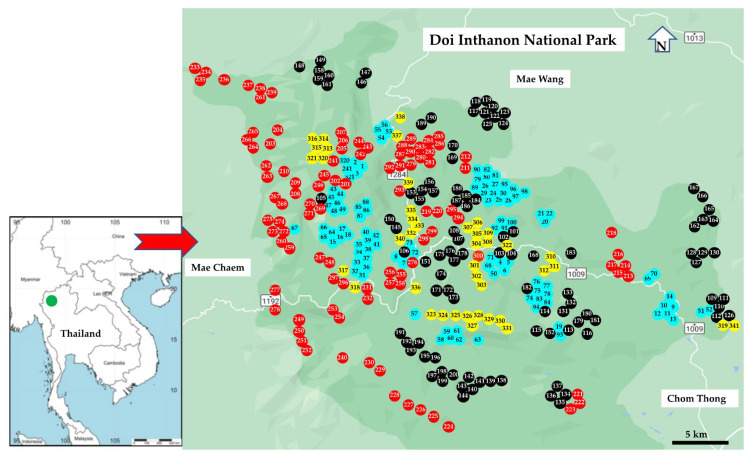
Map of Thailand showing the study area (http://www.simplemappr.net, accessed on 20 July 2022) and collection sites in Doi Inthanon National Park; numbers in circles indicate collection numbers (Table S1); blue, 1–100; black, 101–200; red, 201–300; and yellow, 301–341.

Specimens were collected in Doi Inthanon National Park, Chiang Mai Province, northern Thailand (Figure 1). We use the seasonal classification of the Thai Meteorological Department, i.e., rainy season (May–October), cold season (November–February), and hot season (March–April) [66]. 

### 2.2. Mosquito Collections 

The immature stages of mosquitoes (larvae and pupae) were collected monthly from various aquatic habitats from August 2004 to December 2005 using standard dipping and sucking methods. The larvae collected from each site were kept in Whirl-pack bags (width 7 cm, length 17 cm, BioQuip Products, Inc., Compton, CA, USA) containing 100 ml of water from the habitat. The collections were transported to the laboratory and the immature stages of each were reared in individual aluminum bowls. The third- and fourth-instar larvae were separated into individual plastic tubes (2.9 × 8.3 cm) and reared to obtain adult specimens. Emerged adults were pinned, and their associated larval and/or pupal exuviae, along with some fourth-instar larvae from the same collection, were mounted on microscopic slides. Some specimens were also preserved in 75% ethanol.

### 2.3. Larval Mosquito Habitats

During field surveys, each larval habitat was classified as belonging to one of three types of ecosystems, i.e., forest, village, and agriculture. The specific habitats were classified into 31 types in two categories, natural aquatic habitats (animal footprint, bamboo stump, banana stump, bog, cave pool, rock hole, tree hole, ground pool, rice field, etc.) and artificial (human-made) container habitats (bottle, cement tank, jar, tire, etc.) (Table S2). Details of each habitat type were described in Rattanarithikul et al. [23]. 

### 2.4. Mosquito Identification

Morphological identification to species level was performed using the keys for the mosquitoes of Thailand [14,22,23,67,68,69]. Generic classification and abbreviations follow Wilkinson et al. [70,71] and Harbach [72]. Voucher specimens are deposited in the Entomology Section, Queen Sirikit Botanic Garden (QSBGE), Chiang Mai Province, Thailand.

### 2.5. Statistical Analyses

Species richness and abundance of the mosquitoes were recorded for each habitat type. Percentages of relative abundance of each habitat type were calculated by dividing the total number of species that were found in the habitat by the total number of specimens collected.

The percentage of species occurrence (%SO) was calculated by dividing the number of habitat types where a species was taken by the total number of habitat types (*n* = 31). Diversity index analyses for all specimens collected are Shannon_H, Simpson_1-D, Dominance_D, and Evenness_e^H/S. Detrended correspondence analysis (DCA) was used to analyze community structure and distribution of the mosquito species associated with each habitat type. All data were analyzed using PAST version 4.07 [73]. To compare mosquito diversity with different sample sizes in each community and sampling sufficiency, species accumulation rarefaction and extrapolation curves were generated using the iNEXT software (Online Version 2022). Comparisons between the natural habitats and artificial containers (human-made), and between habitat types (forest, agriculture area, and village) and seasonal diversity were carried out with 1000 randomizations without replacement and at a 95% confident interval [74].

## 3. Results

### 3.1. Species Diversity and Community Structure

**Figure 2:**
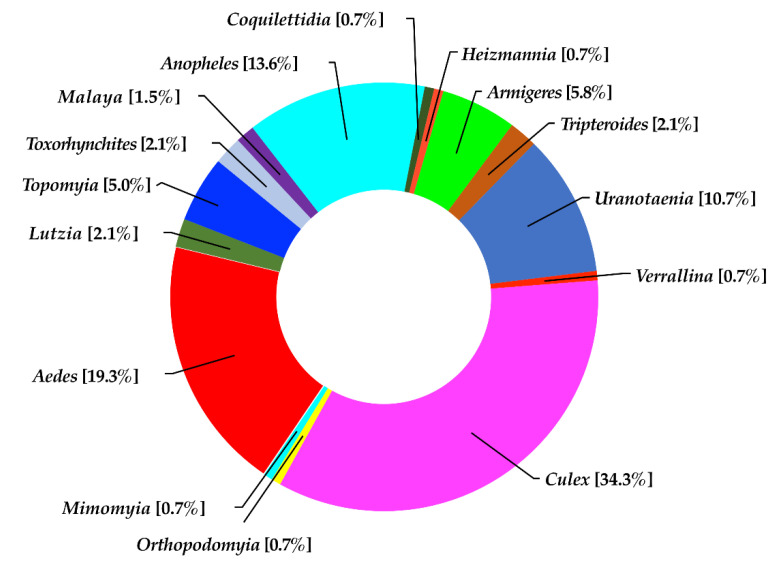
Community structure of mosquitoes in Doi Inthanon National Park.

A total of 3795 specimens comprising 140 mosquito species in 15 genera of the two subfamilies (Anophelinae and Culicinae) were identified from 341 collection sites in Doi Inthanon National Park (Table S2). Five dominant genera, *Culex* (34.3% of the specimens), *Aedes* (19.3%), *Anopheles* (13.6%), *Uranotaenia* (10.7%), and *Armigeres* (5.8%), were represented by 48, 27, 19, 15, and 8 species, respectively. Only one species was found in each of the genera *Coquillettidia*, *Heizmannia*, *Mimomyia*, *Orthopodomyia*, and *Verrallina* (Figure 2). 

Among the 140 species collected, the most relatively abundant species were *Aedes albopictus* (6.7%), followed by *Tripteroides aranoides* (5.6%), *Culex mimulus* (5.0%), *Cx. bitaeniorhynchus* (4.3%), and *Ae. vittatus* (3.8%). *Aedes harveyi* and *Cx*. *mimulus* had the highest percentage of occurrence (52%), followed by *Ae*. *albopictus* (45%), *Cx. sasai*, and *Cx.* sp. 4 (36%) (Table S2).

Of the 140 species, 10 (7.2%) were each represented by more than 100 specimens. In total, 8 species (5.7%) included 51 to 100 specimens, and 57 species (40.7%) included 10 to 50 specimens, while 65 species (46.4%) were represented by fewer than 10 specimens, and 14 species were represented by only one or two specimens (Table S2). 

**Figure 3:**
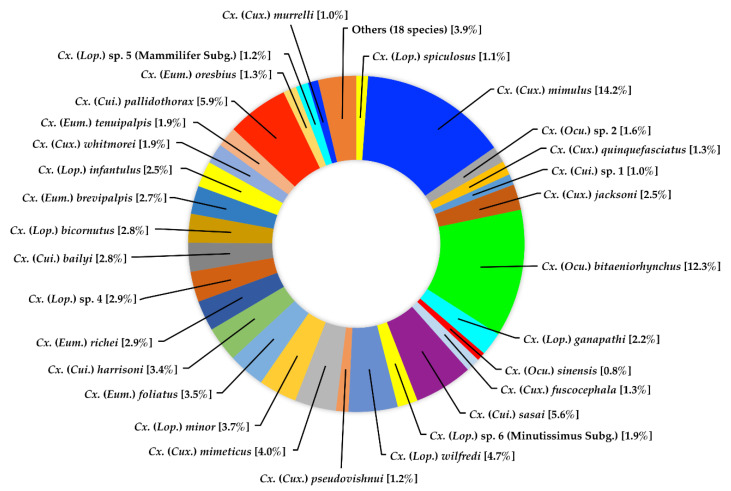
Species composition and abundance of mosquitoes of the genus *Culex* collected in Doi Inthanon National Park.

Species richness and abundance of four main genera, *Culex*, *Aedes*, *Anopheles*, and *Uranotaenia*, are described sequentially. For *Culex*, 48 species were identified from 1334 specimens. *Culex mimulus* was the most abundant species, which represented 14.2% of the total number of specimens collected, followed by *Cx*. *bitaeniorhynchus* (12.3%), *Cx*. *pallidothorax* (5.9%), *Cx*. *sasai* (5.6%), and *Cx*. *wilfredi* (4.7%) (Figure 3).

**Figure 4:**
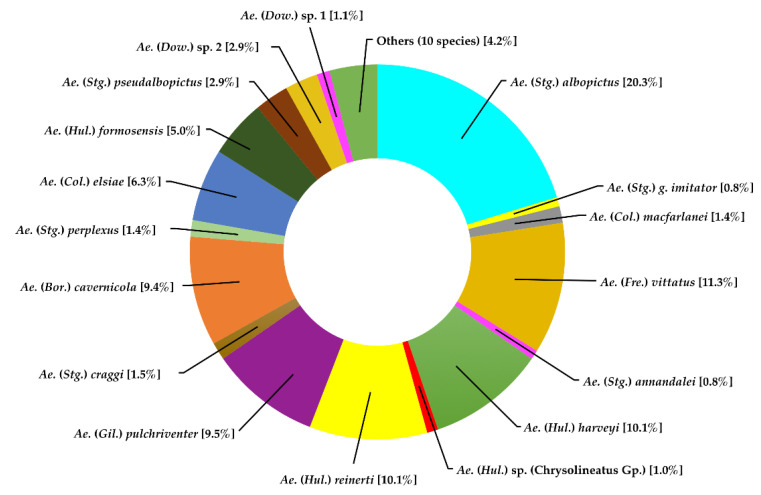
Species composition and abundance of mosquitoes of the genus *Aedes* collected in Doi Inthanon National Park.

Twenty-seven species belonging to 10 subgenera of the genus *Aedes* were identified from 1262 specimens. *Aedes* (*Stegomyia*) *albopictus* was the most abundant species, which represented 20.3% of the total number of *Aedes* specimens collected, followed by *Ae*. (*Fredwardsius*) *vittatus* (11.3%), *Ae*. (*Hulecoeteomyia*) *harveyi* (10.1%), *Ae.* (*Hul*.) *reinerti* (10.1%), and *Ae*. (*Gilesius*) *pulchriventer* (9.5%) (Figure 4).

**Figure 5:**
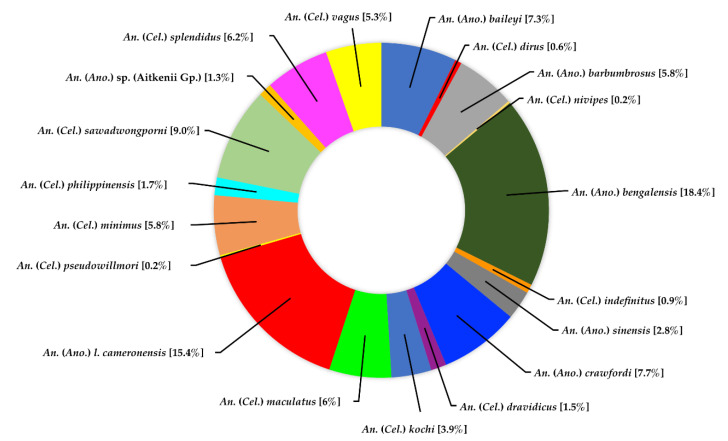
Species composition and abundance of mosquitoes of the genus *Anopheles* collected in Doi Inthanon National Park.

Genus *Anopheles*. A total of 467 specimens were identified, comprising 19 species in two subgenera (*Anopheles* and *Cellia*). *Anopheles (Anopheles*) *bengalensis* was the most abundant species, accounting for 18.4% of all recorded *Anopheles* specimens, followed by *An*. (*Ano*.) *cameronensis* (15.4%), *An*. (*Cellia*) *sawadwongporni* (9.0%), *An*. (*Ano*.) *crawfordi* (7.7%), and *An*. (*Ano*.) *baileyi* (7.3%) (Figure 5).

**Figure 6:**
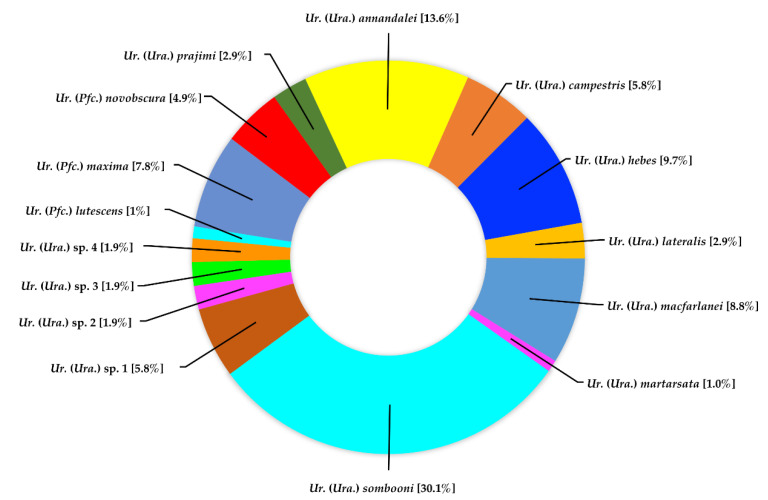
Species composition and abundance of mosquitoes of the genus *Uranotaenia* collected in Doi Inthanon National Park.

Genus *Uranotaenia*. A total of 103 specimens were identified, comprising 15 species in two subgenera (*Uranotaenia* and *Pseudoficalbia*). *Uranotaenia (Uranotaenia*) *sombooni* was the most abundant species, representing 30.1% of all *Uranotaenia* specimens collected, followed by *Ur.* (*Ura*.) *annandalei* (13.6%), *Ur*. (*Ura*.) *hebes* (9.7%), and *Ur*. (*Ura*.) *macfarlanei* (8.8%) (Figure 6).

Genus *Armigeres*. Eight species of *Armigeres* were identified. *Armigeres* (*Leicesteria*) *flavus* was the most abundant species, accounting for 61.0% of all specimens of *Armigeres* collected, followed by *Ar.* (*Armigeres*) *subalbatus* (13.9%) and *Ar.* (*Lei.*) *magnus* (9.1%). 

Genus *Lutzia*. Three species in the genus *Lutzia* were collected. *Lutzia chiangmaiensis* was the most abundant, representing 64.7% of the *Lutzia* identified, followed by *Lt*. (*Metalutzia*) *vorax* (20.6%) and *Lt.* (*Mlt.*) *fuscana* (14.7%). 

Genus *Topomyia*. Seven species of *Topomyia* were identified. The three most abundant species were *To*. spp. 1 (31.5%), *To.* (*Topomyia*) *lindsayi* (23.6%), and *To.* (*Top*.) sp. 2 (18.0%). 

Other genera. Data for seven other genera, for which relatively few specimens were collected, including *Coquillettidia*, *Heizmannia*, *Mimomyia*, *Orthopodomyia*, *Toxorhynchites*, *Tripteroides*, and *Verrallina*, are provided in Table S2.

### 3.2. Diversity Comparisons

**Figure 7:**
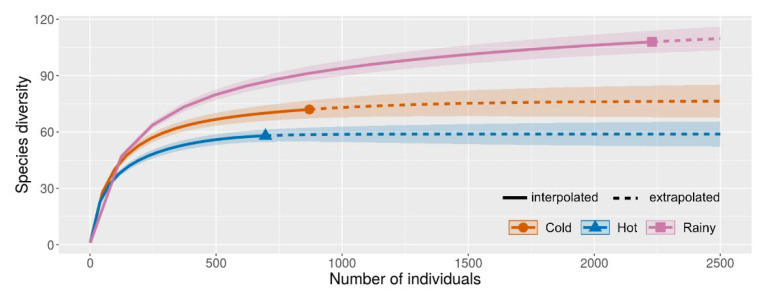
Mosquito species richness accumulation curves showing seasonal richness comparisons. The shaded area on either side of the lines represents 95% confidence intervals. Solid line = interpolated; dash line = extrapolated. Estimated sample coverage of the reference sample for each season: rainy = 0.9928, cold = 0.9908, and hot = 0.9943. q = 0, species richness with bootstrap of 1000.

Seasonal species richness and abundance of mosquitoes revealed distinct differences between seasons, with peaked abundance in the rainy season, followed by the cold and hot seasons (Figure 7).

Sample coverage of each season was high in all seasons (rainy = 0.993, cold = 0.991, hot = 0.994), indicating that the sampling in this study was efficient. Diversity data of mosquitoes collected during each season are shown in Table 1. A total of 32 species (23% of all species collected) were encountered throughout the year, whereas 74 species (53%) were found in only one season, in which their activity was dependent. Interestingly, the most relatively abundant species, *Ae*. (*Stegomyia*) *albopictus*, was found during the rainy (peak) and cold seasons but was absent in the hot season. 

**Figure 8:**
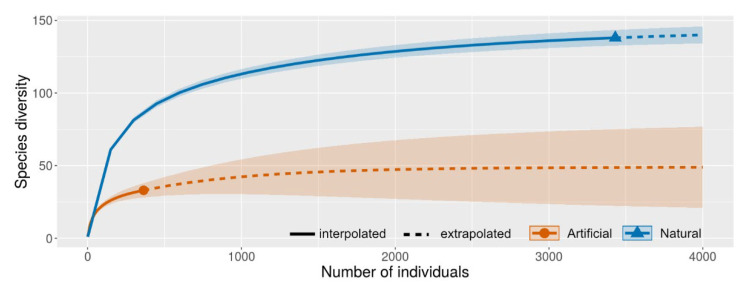
Diversity comparisons for mosquitoes in natural habitats and artificial containers. The shaded area represents 95% confidence intervals. Solid line = observed species (interpolation); dash line = extrapolation species richness. Sample coverage of the reference sample for each season: natural = 0.996, artificial = 0.986. q = 0, species richness with bootstrap of 1000.

Species diversity of mosquitoes in natural and artificial containers is compared in Table S2. The accumulation species richness curve indicated that the number of mosquito species in natural habitats (138) was significantly higher than in artificial containers (33) (t = 23.30, df = 442.620, *p* < 0.001) (Figure 8). The diversity data for mosquitoes in both natural and artificial habitats are presented in Table 1.

**Figure 9:**
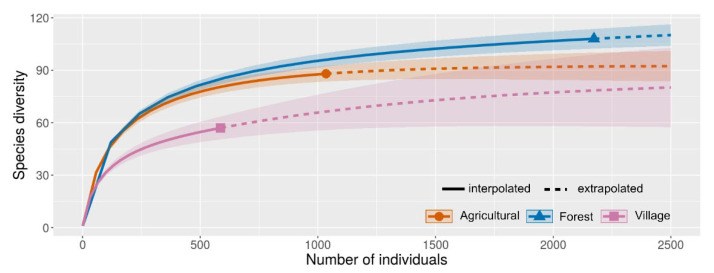
Diversity comparisons for mosquitoes in forest, agriculture, and village areas. The shaded area represents 95% confidence intervals. Solid line = observed species (interpolation); dash line = extrapolation species richness. Sample coverage of the reference sample for each area: forest = 0.993; agriculture = 0.990, and village = 0.975. q = 0, species richness with bootstrap of 1000.

Mosquito diversity in forest, agriculture, and village areas was compared. The species richness accumulation curve revealed that the forest area (108) had significantly more mosquito species than the agriculture (88) and village (57) areas (F = 4.125, df = 151.2, *p* = 0.018) (Figure 9). The diversity data for mosquitoes in each community are shown in Table 1.

### 3.3. Aquatic Habitat Preferences

**Figure 10:**
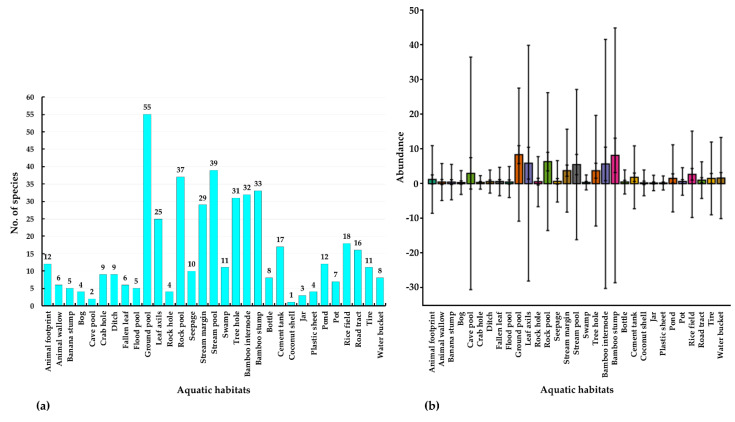
(**a**) Number of species, and (**b**) abundance in aquatic habitats.

The most preferred natural habitats for mosquito species were ground pools, stream pools, and rock pools, which supported 55, 39, and 37 species, respectively. Cement tank, tire, bottle, and water-bucket sites were the most preferred artificial habitats, supporting 17, 11, 8, and 8 species, respectively (Figure 10, Table S2). The diversity index (Shannon_H index) was highest for the ground pool habitats, followed by the rock pool, stream margin, stream pool, and tree-hole habitats, with diversity indices of 3.47, 3.09, 2.94, 2.93, and 2.74, respectively (Table S2). 

### 3.4. Correlation of Mosquito Species with Habitat Types

**Figure 11:**
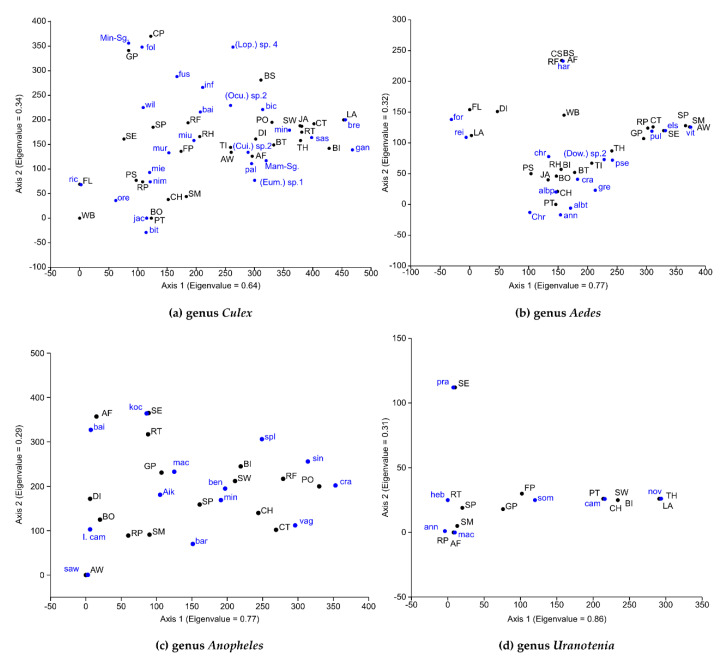
Multivariate ordination of detrended correspondence analysis (DCA) of mosquito species of four dominant genera which correlated to habitat types in Doi Inthanon National Park. (**a**) *Culex*, (**b**) *Aedes*, (**c**) *Anopheles*, and (**d**) *Uranotaenia*. Blue letters = mosquito species; black letters = habitat type. Abbreviations of mosquito species and types of habitats are given in Table S2.

Detrended correspondence analysis indicated that the distribution of species of each genus of mosquitoes correlated with habitat types (Figure 11). Mosquitoes of the genus *Culex* were found in 93.5% (29/31) of all habitat types. Most *Culex* species were associated with ground pools, stream pools, rock pools, stream margins, and bamboo stumps, which harbored 29, 19, 17, 16, and 11 species, respectively (Figure 11a). The most widely distributed and abundant species, *Cx*. (*Culex*) *mimulus*, was positive in 16 habitat types, with high abundance observed in ground pools, rock pools, and stream pools, but it was also found in a wallow, leaf axils, a swamp, and a jar. *Culex (Culiciomyia) sasai* was found in 35.5% (11/31) of all habitat types, with high abundance in cement tanks and fallen leaves, but it was also found in stream pools and bamboo internodes. *Culex* (*Lophoceraomyia*) sp. 4 was found in 35.5% of all habitat types, with high abundance in the rock pool and ground pool habitats. Additionally, some specimens of this species were found in tree holes, bamboo stumps, and plastic sheets. *Culex* (*Oculeomyia*) *bitaeniorhynchus* was found in 23.6% (7/31) of all habitat types, the occurrence of which was strongly correlated with stream pool and rice field habitats. The medically important species *Cx. (Cux.*) *quinquefasciatus* was found only in artificial container habitats (tires and cement tanks). The remaining 15 *Culex* species were restricted to a single habitat type. For example, *Cx.* (*Cui*.) *harrisoni* was found only in a cave pool, *Cx.* (*Cux*.) *barraudi* in ground pools, and *Cx*. (*Ocu*.) *sinensis* in rice fields (Table S2).

Mosquitoes of the genus *Aedes* were found in 83.9% (26/31) of all habitat types, and most of those species were found in bamboo internodes, tree holes, and stream pools (Figure 11b). The most encountered species (most occurrence records), *Ae. (Hulecoeteomyia*) *harveyi*, was found in 61.5% (16/26) of all habitat types, with high abundance in water bucket, banana stumps, bamboo internodes, bamboo stumps, and coconut shells. This species was also found in various other habitat types, such as tree holes, cement tanks, stream pools, animal footprints, and crab holes. The most abundant species, *Ae. (Stg.*) *albopictus* (a medically important species), was found in 53.8% (14/26) of all habitat types. High numbers of this species were collected from the bamboo stumps, bamboo internodes, tires, rock holes, and a water bucket. The remaining five species of *Aedes* collected were specific to various habitat types, such as cave pool for *Ae.* (*Borichinda*) *cavernicola*, tree hole for *Ae*. (*Downsiomyia*) *albolateralis*, and bamboo stump for *Ae*. (*Dow.*) *novoniveus* (only one specimen) (Table S2).

Mosquitoes of the genus *Anopheles* were found in 51.6% (16/31) of all habitat types, and most species were found in ground pools, stream pools, and rice fields (Figure 11c). *Anopheles* (*Ano*.) *cameronensis* was found in the greatest number of habitat types, having been found in 50% (8/16) of all types. The highest density of this species was recorded in ground pools, stream margins, ditches, stream pools, and a bog located at high elevation (2520 m above sea level). *Anopheles* (*Ano*.) *bengalensis* was the most abundant species, which was found in 7/16 (43.8%) of all habitat types. It was strongly associated with stream pools, ground pools, and stream margins. Among malaria vectors in Thailand, *An*. (*Cel.) minimus* was found in stream pools, stream margins, and ground pools in high densities, and some specimens were found in crab holes, swamps, and rice fields. The great numbers of *An.* (*Cel.*) *maculatus* were recorded in ground pools and rock pools, and one specimen was found in a bamboo internode. Most specimens of *An.* (*Cel*.) *sawadwongporni* were found in rock pools and stream margins but they were also found in a cement tank. *Anopheles* (*Cel*.) *dirus* was only found in animal footprints. Only one specimen each of *An.* (*Cel*.) *nivipes*, *An.* (*Cel*.) *pseudowillmori*, and *An.* (*Cel*.) *vagus* was found in a ground pool (Table S2).

Members of the genus *Uranotaenia* were found in 48.4% (15/31) of all habitat types, and most species were collected from ground pools, rock pools, and stream pools (Figure 11d, Table S2). *Uranotaenia* (*Ura*.) *sombooni* was the most widely distributed species, found in ground pools and flood pools. Some specimens were found in a stream pool and stream margin, and one specimen was found in a bamboo internode. *Uranotaenia* (*Ura.*) *annandalei* was found in a stream margin and stream pool.

Six species of the genus *Topomyia* were collected from leaf axils, while some were found in fallen leaves, tree holes, and bamboo internodes. Species of the genus *Toxorhynchites* were mostly found in bamboo stumps, bamboo internodes, tree holes, and bottles. The most abundant species, *Tx.* (*Toxorhynchites*) *gravelyi*, was found in bamboo stumps, while *Tx*. (*Tox.*) *splendens* was found only in tree holes and *Tx*. (*Tox*.) *leicesteria* only in bamboo internodes. Three species of the genus *Tripteroides* were found in bamboo internodes, bamboo stumps, and tree holes but most were found in bamboo internodes. 

The species of six genera for which the fewest specimens were collected were each restricted in only one habitat type. For instance, *Cq.* (*Coquillettidia*) *crassipes* were found only in ponds, *Hz*. (*Heizmannia*) *reidi* and *Or. anopheloides* in tree holes, *Ml*. *genurostris* and *Ml. jacobsoni* in leaf axils, one specimen of *Mi. (Eto.*) *luzonensis* in an animal footprint, and *Ver*. sp. 1 in ground pools (Table S2).

## 4. Discussion

The present study indicates that Doi Inthanon National Park is an important area for mosquito diversity. The park has high species richness and abundance of mosquitoes representing one third of the total number of species recorded in Thailand [4]. The topographic complexity and variety of aquatic sources in the park are available for mosquitoes throughout the year [49,57,75]. The great diversity of mosquitoes (140 species in 15 genera) collected in this study is consistent with previous reports. For example, 160 species in 18 genera were found in the Amazon rainforest [76], 117 species in 17 genera in the northwestern Amazon [35], and 103 species in 16 genera in Cantareira State Park in Brazil [77]. However, low numbers of species can be affected by different collection methods, as seen in some previous studies, such as 26 species in five genera collected in Vietnam [78], 50 species in 12 genera in the Amazon basin, Peru [79], 82 species in the Caatinga biome, Brazil [80], 20 species (10,131 specimens) from Amazonas State, Brazil [37], and 103 species in 16 genera in Cantareira State Park, Brazil [77]. According to these data, it is obvious that the tropical region is an area with high levels of mosquito diversity. However, the diversity, distribution, and abundance of mosquitoes are not only dependent on suitable habitats, but also on physiochemical factors in those habitats that influence larval occurrence [7,81,82,83].

Findings for the dominant genus *Culex* in this study correspond with accounts in previous reports [77,79,80]. Most species in this genus have high abundance and richness in forest areas [67,84]. Our results showed that *Culex* larvae were found in various types of habitats, which is similar to the previous findings reported by [7,27,67,85]. Doi Inthanon National Park has environmental heterogeneity that supports a variety of habitats for *Culex* mosquitoes, especially ground pools, rock pools, and rice fields in the rainy season. Similarly, some studies have found that the majority of habitat types utilized by *Culex* are semi-permanent or permanent bodies of fresh, brackish, clean, or heavily polluted water, and several species develop in leaf axils, tree holes, rock holes, crab holes, and other small collections of water [86,87]. As in another study, *Cx.* (*Cux*.) *mimulus*, the most abundant *Culex* species in the present study, was mostly found in ground pools and rock pools, but specimens were occasionally found in other habitats, including an animal wallow, leaf axils, tree holes, and a jar [67]. In addition, our data for *Cx. (Cux.*) *quinquefasciatus* are consistent with the previous report by Almirón and Brewer [88], who also found this species more frequently in artificial containers (tires, tank in disuse, and cistern). The habitats of 27 species of *Aedes* collected in the present study are accordant with those recorded by Rattanarithikul et al. [14,23], which included tree holes, bamboo stumps, and bamboo internodes as the principal habitats for *Aedes* larvae in Thailand. *Aedes* (*Stg*.) *albopictus* occupied the widest variety of habitats, especially phytotelmata and artificial containers, with a higher abundance in natural containers than in artificial containers. This finding agrees with the findings of studies conducted in several countries (Thailand, Brazil, Malaysia, Vietnam, China, and USA) in which *Ae. albopictus* also occurs in both natural and artificial containers [14,89,90,91,92,93,94]. However, those studies were conducted in the rural and suburban areas, and a higher abundance in artificial containers than in natural containers was observed. For example, Vanwambeke et al. [89] found *Ae*. *albopictus* mainly in rural and peri-urban areas, where it was found in natural and artificial containers, with the latter making up 87% of collections. 

We found 19 species of *Anopheles* in Doi Inthanon National Park, approximately one fourth of the 83 species recorded in Thailand [18,19,22,95]. Comparison of our findings with those of other studies confirms the high diversity of *Anopheles* in Doi Inthanon National Park. For example, 23 species were recorded in Indonesia based on collections using animal bait and animal-baited trap nets in three ecosystems, i.e., forest, non-forest, and coastal areas [96], 20 species collected by CDC light trap without bait at the China–Myanmar border [34], and 9 and 10 species collected by standard dipping in the Himalayan region and Ethiopia, respectively [7,97]. The larval habitats for species of this genus were strongly associated with a variety of forest types and clean water [22]. Most *Anopheles* larvae were found in ground pools, stream pools, and rice fields, which is in agreement with previous studies that revealed the typical habitats of the immature stages of anopheline species, which are usually found in clean, fresh, still, or slow-moving waters, and groundwater with abundant floating vegetation. However, some species are restricted to plant cavities in egg-cup-size pools in primary forests in northern Vietnam [98,99]. In a previous study [100], *An*. (*Ano*.) *bengalensis*, the most abundant *Anopheles* species in the present study, was found in a shaded pool beside a stream in a hilly and mountainous area similar to our finding of this species in Doi Inthanon National Park, where the species was mostly found in ground pools, stream pools, and stream margins. Additionally, this common species has been found in several types of habitats in natural forests, including cave holes, rock pools, crab holes, and animal footprints [22]. *Anopheles* (*Ano.*) *cameronensis* was mostly found in ground pools, stream margins, and bog in forest areas at elevations ranging between 1200 m and the summit of Doi Inthanon (2500 m), as reported by other researchers [22,100]. However, the dominance and abundance of mosquito species varied depending on collection methods and in different ecosystems [7,40,46,48,96]. Interestingly, one specimen each of *An*. (*Ano*.) *sinensis* and *An*. (*Cel*.) *maculatus* was found in a bamboo internode despite commonly occurring in rice fields, grassy ponds, ditches, springs, seepages, and small streams partly exposed to sunlight [99]. Therefore, changes in environment and ecology, and the effect of human activities are affecting the mosquito community, leading to adaptation to new aquatic habitats. Thus, regular entomological evaluation is essential for vector monitoring.

Based on the results of the present study, the seasonal diversity and abundance of mosquitoes in Doi Inthanon National Park varies depending on the species, some of which are present throughout the year. However, in general, species richness and abundance peak in the rainy season and decrease in the cold and hot seasons, in agreement with the finding of other studies conducted in the Himalayan region [7], Ghana [101], and Mexico [102]. Several studies conducted on vector species in Thailand have shown that species richness and populations exhibit high fluctuation in the rainy season [39,42,43,47]. Rainfall is the key factor that influences the diversity and distribution of mosquitoes. 

While comparing the diversity of mosquito species in natural and artificial habitats, it was discovered, as expected, that natural habitats support many more species than artificial habitats. Based on a comparison of the findings of this study with those of previous studies, it is obvious that there is a high diversity of species in forests, as opposed to low diversity but higher abundance in areas outside of forests, particularly in artificial habitats [37,96,103]. Doi Inthanon National Park is primarily comprised of forested areas that provide a variety of habitats for various mosquito species, whereas artificial habitats are generally found in villages, agricultural areas, and some tourist sites, which are minor areas of the park and are unsuitable for many species other than *Ae*. (*Stg*.) *albopictus*, which is abundant in such areas.

## 5. Conclusions

Based on the results of this study, it is apparent that Doi Inthanon National Park has very diverse populations of mosquitoes. *Culex* species and *Ae*. (*Stg*.) *albopictus* are present in highest abundance. Species richness is highest in the rainy season, much lower in the cold and hot seasons, and natural habitats harbor more species than artificial habitats. Forests host a greater diversity of species than agricultural and village areas. Ground pools, stream pools, and rock pools are important habitats for the immature stages of mosquitoes in the park. Information on the distributions and seasonal activity of mosquitoes obtained in this study is important for planning programs for the control of vector species.

## Figures and Tables

**Table 1:** Diversity of mosquitoes in Doi Inthanon National Park.

Diversity	Season			Aquatic Habitats		Community		
	Hot	Rainy	Cold	Natural	Artificial	Forest	Agriculture	Village
Species richness	72	108	58	138	33	108	88	57
Abundance	696	2229	870	3431	364	2173	1035	587
Shannon_H	3.55	3.81	3.72	4.20	2.82	3.97	3.81	3.21
Dominance_D	0.040	0.039	0.037	0.023	0.094	0.028	0.036	0.072
Simpson_1-D	0.960	0.961	0.963	0.977	0.906	0.972	0.964	0.928
Evenness_e^H/S	0.598	0.419	0.574	0.483	0.506	0.488	0.514	0.436
Equitability_J	0.873	0.814	0.870	0.852	0.806	0.847	0.852	0.795

## Data Availability

The data presented in this study are available in the Supplementary Materials.

## Supplementary Materials

The following supporting information can be downloaded at: https://www.mdpi.com/article/10.3390/insects13090814/s1, Table S1: Collection sites of mosquitoes in Doi Inthanon National Park, Chiang Mai, Thailand. Table S2: Species lists of mosquitoes collected in Doi Inthanon National Park.
